# Gearbox Fault Diagnosis Based on Adaptive Variational Mode Decomposition–Stationary Wavelet Transform and Ensemble Refined Composite Multiscale Fluctuation Dispersion Entropy

**DOI:** 10.3390/s24227129

**Published:** 2024-11-06

**Authors:** Xiang Wang, Yang Du, Xiaoting Ji

**Affiliations:** 1 School of Energy and Power Engineering, Nanjing Institute of Technology, Nanjing 211167, China; y00450230902@njit.edu.cn; 2 Jiangsu Provincial Key Laboratory of Multi-Energy Integration and Flexible Power Generation Technology, Nanjing 211167, China; 3 School of Electrical Engineering, Nanjing Institute of Technology, Nanjing 211167, China; y00450220746@njit.edu.cn

**Keywords:** fault diagnosis, adaptive variational mode decomposition, fluctuation dispersion entropy, gearbox

## Abstract

Existing gearbox fault diagnosis methods are prone to noise interference and cannot extract comprehensive fault signals, leading to misdiagnosis or missed diagnosis. This paper proposes a method for gearbox fault diagnosis based on adaptive variational mode decomposition–stationary wavelet transform (AVMD-SWT) and ensemble refined composite multiscale fluctuation dispersion entropy (ERCMFDE). Initially, the kurtosis coefficient and autocorrelation coefficient are presented, and the Intrinsic Mode Functions are denoised through the application of AVMD-SWT. Secondly, the coarse-grained processing method of composite multiscale fluctuation dispersion entropy is extended to encompass three additional approaches: first-order central moment, second-order central moment, and third-order central moment. This enables the comprehensive extraction of feature information from the time series, thereby facilitating the formation of an initial hybrid feature set. Subsequently, recursive feature elimination (RFE) is employed for feature selection. Ultimately, the outcomes of the faults diagnoses are derived through the utilization of a Support Vector Machine with a Sparrow Search Algorithm (SSA-SVM), with the actual faults data collection and analysis conducted on an experimental platform for gearbox fault diagnosis. The experiments demonstrate that the method can accurately identify gearbox faults and achieve a high diagnostic accuracy of 98.78%.

## 1. Introduction

Gearboxes are crucial mechanical transmission components that are ubiquitous in a plethora of engineering, aeronautical, automotive, renewable energy, and other industrial fields [1,2]. The financial expenditure incurred for the operation, maintenance, and repair of gearboxes is significant [3]. It is, therefore, of the utmost importance to guarantee the long-term smooth operation of a gearbox in order to ensure the safe operation of a unit and to enhance the operating efficiency of the unit [4,5]. The deployment of fault diagnosis techniques that can provide timely warnings and accurately identify the type of fault is a pivotal step for ensuring the optimal functioning of gearboxes [6,7]. The implementation of precise troubleshooting methodologies can serve to safeguard the normal operation of the gearboxes [8]. The more recent technological developments in the field of fault diagnosis entail the collection of essential operational data from gearboxes, followed by the utilization of sophisticated signal analysis techniques to ascertain the current health status of the gearboxes [9]. This approach enables the precise identification of the underlying fault and the optimization of maintenance operations. For example, J. Yan et al. proposed an unsupervised machine abnormal sound detection model based on a Transformer and Dynamic Graph Convolution (Unsuper-TDGCN), which is a diagnostic method based on faulty equipment sound data [10]. In addition, Y. Cheng et al. proposed a feature-adaptive method called IES via a candidate fault frequency optimization-gram (IESCFFOgram) method based on a candidate fault frequency optimization graph, which is a fault diagnosis method based on vibration signals [11]. In recent years, a vibration signal-based fault diagnosis method has become a widely utilized technique [12]. The technique comprises three distinct phases: signal processing [13], feature extraction [14], and fault identification [15].

A gearbox is constituted of a series of components, including gears, bearings, lubrication system, and housing [16]. Gearboxes utilize the principle of mutual meshing between the gears to facilitate the transmission of power and the conversion of mechanical energy [17]. Gearbox gears are susceptible to a range of failures, including tooth pitting, broken teeth, gear eccentricity, and plastic deformation, as a result of prolonged exposure to high speeds and heavy loads [18]. However, due to the challenging operational conditions of a gearbox, the collected vibration signal exhibits a considerable degree of noise [19]. This makes signal feature extraction more difficult. It is therefore necessary to analyze the signal in the time–frequency domain to reduce noise interference [20]. The commonly used vibration signal processing methods are Fourier transform [21], wavelet transform [22], Wigner–Ville transform [23], Karhunen–Loève transform [24], Singular Spectrum Analysis [25] (SSA), Empirical Mode Decomposition [26] (EMD), Local Mean Decomposition [27] (LMD), variational mode decomposition [28] (VMD), and their improvement methods. The basic principle of VMD is to iteratively find the optimal solution of a constrained variational model to determine the center frequency and bandwidth of each Intrinsic Mode Function (IMF). The separation of the components and decomposition of the signal are achieved by minimizing the sum of the estimated bandwidths of each component through an iterative updating process. Among them, VMD decomposes the signal into a set of IMFs by solving an optimization problem to obtain the optimal IMF. The optimization objective of VMD is to minimize the distance between the signal and the atomic signal while maintaining orthogonality between IMFs. Compared to EMD, VMD has a stronger mathematical foundation and stability. It is not only suitable for nonlinear signal decomposition, but also overcomes endpoint effects and mode-mixing phenomena, and has a stronger noise suppression effect. He et al. [29] used the parameter optimization of VMD to address the nonlinear and non-stationary characteristics in bearing vibration signals. Niaki et al. [30] overcame the challenge of extracting features for early fault detection in gearboxes by combining VMD with time synchronous averaging to extract certain features. However, VMD has some adjustable parameters, and different parameter selections will significantly affect the decomposition results of VMD. Taibi et al. [31] employed a whale optimization algorithm and grey wolf optimization algorithm to find the optimal parameters for VMD. Yang et al. [32] optimized the relevant parameters of VMD using the marine predator algorithm. Traditional optimization algorithms usually require multiple iterations to find the optimal parameters, with high computational complexity and overfitting. Kurtosis determines the validity and information retention of the IMF, while the autocorrelation coefficient reveals the correlation of the signal at different time lags. This paper will introduce kurtosis and autocorrelation coefficients to measure the relationship between the IMF and the original signal, and adaptively select the best decomposition parameters.

The vibration signals of gearboxes often exhibit nonlinear characteristics under actual operating conditions. This implies that nonlinear factors are involved in the generation and propagation of vibration signals, resulting in vibration signals that deviate from the characteristics typically observed in linear systems. The conventional nonlinear signal analysis indices, including the mean, variance, kurtosis, skewness, peak, and waveform factor, are challenging to utilize effectively for the extraction of fault characteristic information. It is, therefore, crucial to determine how to extract the pertinent information from such nonlinear vibration data in order to gain insight into the operational status of the equipment. In light of the aforementioned evidence, the thermodynamic concept of entropy is duly referenced. Entropy is a measure of the complexity of a time series that is commonly used to quantify the level of uncertainty and confusion of the information in a signal. Scholars have introduced the concept of entropy theory into the field of equipment fault diagnosis with the objective of providing a quantitative characterization of the degree of complexity under the statistical significance of a time series. This approach has yielded promising results in practice. The use of a single scale of entropy presents a challenge for accurately characterizing the complexity of a time series, with the potential for erroneous conclusions. Consequently, the concept of multiscale entropy was introduced and subsequently expanded upon within a multiscale framework. To illustrate this, Li et al. [33] put forth a fault diagnosis method that is capable of identifying the disparate fault types of planetary gearboxes through the utilization of an enhanced multiscale symbolic dynamic entropy in conjunction with the Minimum Redundancy Maximum Relevance. Wu et al. [34] extracted the feature vectors from faulty vibration signals using a multiscale permutation entropy and applied Support Vector Machine (SVM) to automate the fault diagnosis process. This approach demonstrated superior performance compared to the methods based on a single-scale permutation entropy and multiscale entropy. Zheng et al. [35] put forth a methodology for the diagnosis of machine faults that employs a multiscale transfer fuzzy entropy and SVM for the identification of diverse fault types. Bing et al. [36] put forth a rolling bearing fault diagnosis technique, namely a multi-class Support Vector Machine, which employs a fine-grained multiscale symbolic entropy and a whale optimization algorithm. Nevertheless, the length of a coarse-grained time series following a multiscale transformation demonstrates a proclivity towards reduction in accordance with the scale factor. This alteration has the direct effect of reducing the stability of the entropy value. Moreover, the majority of the most prevalent algorithms are constrained to the processing of first-order central moments, i.e., mean values, thereby failing to fully leverage the extensive higher-order information embedded in a time series. In light of the aforementioned findings, this paper proposes an innovative enhancement to the feature extraction method.

In the selection of an entropy algorithm, the FDE algorithm is the optimal choice due to its superior accuracy in entropy calculation, enhanced robustness, and reduced susceptibility to noise interference. Consequently, we have selected the FDE as the characteristic entropy to effectively reflect the distinctive features of a fault signal. This paper develops the traditional first-order central moment to encompass the multi-order central distance. This method preserves the traditional first-order central moments as a fundamental aspect while introducing two supplementary dimensions: the variance as the second-order central moment and the skewness as the third-order central moment. This provides a more nuanced representation of the data. This approach not only enhances the level and depth of the feature space but also facilitates the capture of the volatility and skewness of the distribution pattern in the time series data, thereby enabling a more comprehensive and detailed description of the features of a time series. The processing of these extracted features from disparate dimensions and perspectives in parallel enables the construction of a more three-dimensional and multidimensional time series feature representation system. The parallel processing mechanism not only improves the efficiency of feature extraction, but also enhances the model’s ability to capture the complex dynamic changes in a time series. This provides a robust foundation for data analysis and fault prediction.

In light of the advantages offered by the AVMD-SWT model, which exhibits superior complex data decomposition accuracy and enhanced resilience to noise interference, and the ERCMFDE approach, which provides a comprehensive extraction of features from diverse dimensions and perspectives, an intelligent diagnosis method for gearbox faults based on AVMD-SWT and ERCMFDE is proposed. The main contributions of this paper can be summarized as follows:

1. The proposed AVMD-SWT denoising model, which introduces kurtosis and autocorrelation coefficients to adaptively determine the number of decomposition layers in the VMD, and utilizes stationary wavelet transform to denoise the mode components containing noise.
2. It addresses the issues of inaccurate and incomplete feature extraction in traditional multiscale entropy algorithms for the feature extraction of vibration signals by proposing an ensemble refined composite multiscale fluctuation dispersion entropy algorithm based on multi-order central moments.
3. An intelligent fault diagnosis model for a gearbox is constructed based on AVMD-SWT, ERCMFDE, RFE, and SSA-SVM.
4. The efficacy of the model is validated through the simulation of gearbox operational states on the MFS experimental platform. The results demonstrate that the ERCMFDE outperforms the single refined composite multiscale fluctuation dispersion entropy (RCMFDE) in terms of its comprehensive feature extraction and higher fault diagnosis accuracy.

## 2. Signal Processing and Feature Extraction Theory

### 2.1. AVMD-SWT

#### 2.1.1. Variational Mode Decomposition

VMD is a commonly used method in signal decomposition, which decomposes signals by minimizing the differences between a signal and each mode. It can decompose signals into a set of steady-state vibration modes, solving the mode-mixing problem inherent in EMD. VMD is a signal decomposition method based on a time–frequency domain analysis. By partitioning the frequency domain of a signal, it decomposes the signal into K IMFs with a specific sparsity, where K represents the total number of components. Each IMF has a signal bandwidth distributed around its central frequency. The principle of VMD can be divided into constructing the variational problem and solving it [37].

Constructing the variational problem: The K IMFs are decomposed from the original signal, where the *i*th IMF can be represented as

(1) $$u_{i}(t)=A_{i}(t)\cos(\phi_{i}(t))$$

where $A_{i}(t)$ represents the instantaneous amplitude of the *i*th IMF, and $\phi_{i}(t)$ represents the phase angle.

First, perform the Hilbert transform on $u_{i}(t)$ to obtain the spectrum $F_{i}$ in the complex domain

(2) $$F_{i}=(\delta (t)+\frac{j}{t\cdot \pi })*u_{i}(t)$$

where δ(t) is the pulse function.

Next, use a modification function to map each IMF component onto its corresponding fundamental frequency band

(3) $$H_{i}=[(\delta (t)+\frac{j}{t\cdot \pi })*u_{i}(t)]e^{-j\omega_{i}t}$$

where $\omega_{i}$ is the central frequency of the *i*th IMF.

Finally, compute the square of the norm of the demodulated signal *L*^2^, calculate the bandwidth of each IMF, and establish a constrained variational model as follows:

(4) $$\left\{ \begin{array}{l} \underset{\left\{u_{i}\right\},\left\{\omega_{i}\right\}}{\min}\left\{{\sum_{i=1}^{K}\left\|\partial_{t}[(\delta (t)+\frac{j}{t\cdot \pi })*u_{i}(t)]e^{-j\omega_{i}t}\right\|}_{2}^{2}\right\}\\ s.t\sum_{i=1}^{K}u_{i}=f(t) \end{array}\right.$$

where *K* represents the number of decompositions, and f(t) is the original signal.

Solving the variational problem: First, introduce the Lagrangian function to transform the constrained model into an unconstrained one

(5) $$\begin{array}{l} L(\{U_{i}\},\{\omega_{i}\},\lambda )\\ =\alpha {\sum_{i=1}^{K}\left\|\partial_{t}[(\delta (t)+\frac{j}{t\cdot \pi })u_{k}(t)]e^{-j\omega_{i}t}\right\|}_{2}^{2}\\ +\|f(t)-\sum_{i=1}^{K}u_{i}(t){\|}^{2}+\langle \lambda (t),f(t)-\sum_{i=1}^{K}u_{k}(t)\rangle \end{array}$$

where λ is the Lagrange multiplier.

The alternating direction method of multipliers algorithm is used to solve the unconstrained variational problem. The iterative formulas for the parameters are transformed into the complex domain to obtain the update formulas as follows:

(6) $${\hat{u}}_{i}^{n+1}(\omega )=\frac{\hat{f}(\omega )-\sum_{j\neq i}{\hat{u}}_{j}(\omega )+\frac{\hat{\lambda }(\omega )}{2}}{1+2\alpha {\left(\omega -\omega_{k}\right)}^{2}}$$

(7) $${\hat{\lambda }}_{i}^{n+1}(\omega )={\hat{\lambda }}^{n}(\omega )+\gamma (\hat{f}(\omega )-\sum_{i}{\hat{u}}_{i}^{n+1}(\omega ))$$

(8) $$\omega_{i}^{n+1}=\frac{\int_{0}^{\infty }\omega {\left|{\hat{u}}_{i}(\omega )\right|}^{2}d\omega }{\int_{0}^{\infty }{\left|{\hat{u}}_{i}(\omega )\right|}^{2}d\omega }$$

where ${\hat{u}}_{i}^{n+1}(\omega )$ represents the Fourier transform of $u_{i}^{n+1}(\omega )$.

#### 2.1.2. AVMD-SWT Denoising Algorithm

**Figure 1:**
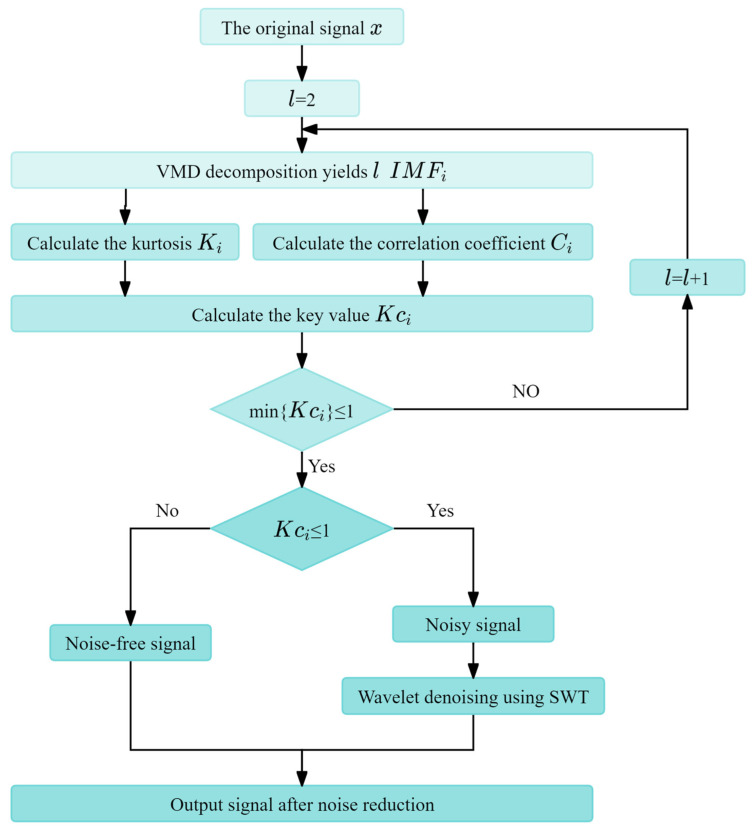
The flowchart of AVMD-SWT.

AVMD-SWT is a two-stage framework denoising algorithm designed for one-dimensional signals. By introducing kurtosis and a correlation coefficient to adaptively select the decomposition parameters of the VMD, and combining these with the advantages of SWT for signal processing, the AVMD-SWT denoising algorithm is proposed. AVMD-SWT plays an important role in signal noise reduction processing, the enhancement of signal characteristics, and the shielding of redundant signal interference by virtue of its adaptive nature and ability to provide a multiscale analysis. Figure 1 shows the flowchart of AVMD-SWT, and the specific implementation steps are as follows [38,39]:

Step 1. VMD: use VMD to decompose the noisy signal, obtaining a set of IMFs, with an initial value of 2 for the decomposition levels.

Step 2. Compute the key values of the mode components: First, calculate the kurtosis $K_{i}$ and correlation coefficient $C_{i}$ of each mode component. Then, use the product of the kurtosis and each mode component as the key value $Kc_{i}$ of each mode component. If the minimum key value is less than 1, it indicates that the skewness and correlation of the signal components are small, and stationary wavelet denoising can be performed. Otherwise, increase the decomposition level by one and continue the VMD.

(9) $$K_{i}=\frac{E{\left(x-\mu \right)}^{4}}{\sigma^{4}}$$

(10) $$C_{i}=\frac{\sum_{j=1}^{N}v_{d}(j,i)\cdot x(j)}{\left\|v_{d}(:,i)\right\|\cdot \left\|x\right\|}$$

(11) $$Kc_{i}=\mathrm{sign}(C_{i})\cdot K_{i}\cdot |C_{i}|$$

where $v_{d}(:,i)$ represents the *ith* component, ‖·‖ represents the magnitude of the vector, and $sign(C_{i})$ represents the sign of the correlation coefficient $C_{i}$.

Step 3. Select the denoised signal components: calculate the key value for each mode component, find the position with the maximum change in the key value, and consider the components with smaller key values as the signal-containing noise components.

Step 4. Initialize the parameters and iteratively optimize: First, initialize the signal length and wavelet decomposition of the signal by a db4 wavelet basis function. Then, calculate the noise standard deviation to prepare for subsequent threshold calculations. Next, select a suitable threshold to perform soft thresholding on the wavelet coefficients, and smooth the signal using L1 regularization constraints [40]. During the iteration process, continuously update the estimated signal components until the stopping criterion is met.

Step 5. Output the denoised signal.

#### 2.1.3. Simulated Signal Analysis

**Figure 2:**
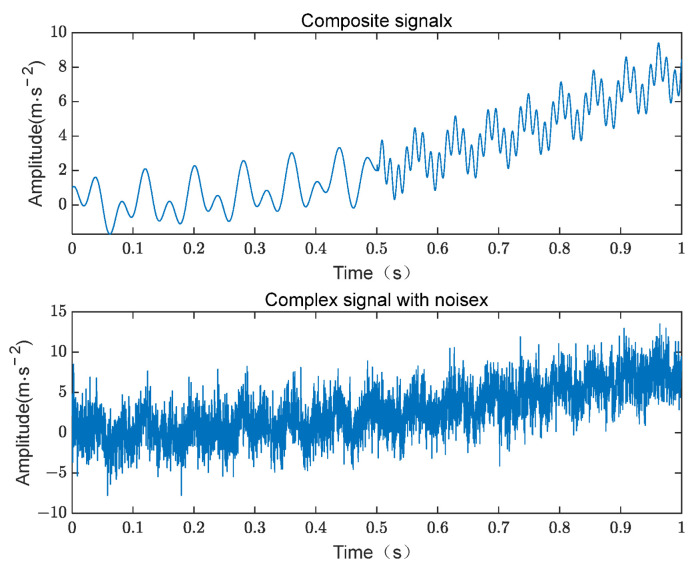
The original composite signal and the composite signal with noise.

This section constructs a piecewise composite signal.

(12) $$x=x_{1}+x_{2}+x_{3}+x_{4}$$

(13) $$\left\{ \begin{array}{l} x_{1}=8t^{3}\\ x_{2}=\sin(2\pi t+10\pi t^{2})\\ x_{3}=\cos(50\pi t),t\leq 0.5\\ x_{4}=\cos(150\pi t-18\pi ),t\geq 0.5 \end{array}\right.$$

where $x_{1}$ represents a signal with a cubic trend, $x_{2}$ denotes a sinusoidal frequency-modulated signal, $x_{3}$ is a cosine signal with a frequency of 50 Hz, and $x_{4}$ is a cosine signal with a frequency of 150 Hz. Simultaneously, 5 dB Gaussian white noise is added to the composite signal for interference. The original composite signal and the composite signal with noise are shown in Figure 2.

**Figure 3:**
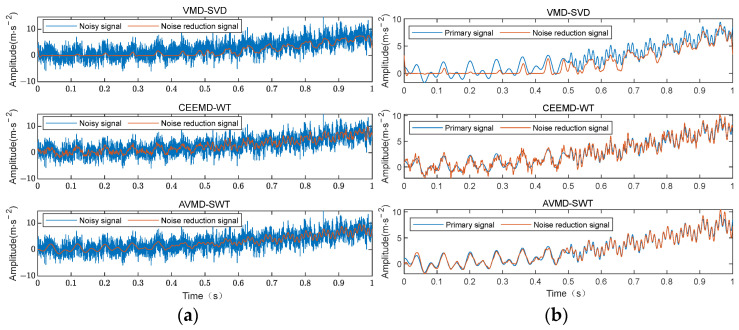
(**a**) Noise reduction signal and noise-containing signal and (**b**) noise reduction signal and original signal.

Using AVMD-SWT, VMD and Singular Value Decomposition (VMD-SVD) [41], and Complementary Ensemble Empirical Mode Decomposition and wavelet transform (CEEMD-WT) [42], the signals are denoised separately. As illustrated in Figure 3a, the comparative analysis of the denoised signals following the three denoising methods and the noisy signals is presented. Figure 3b depicts the comparative analysis of the denoised signals following the three denoising methods and the original signals.

**Figure 4:**
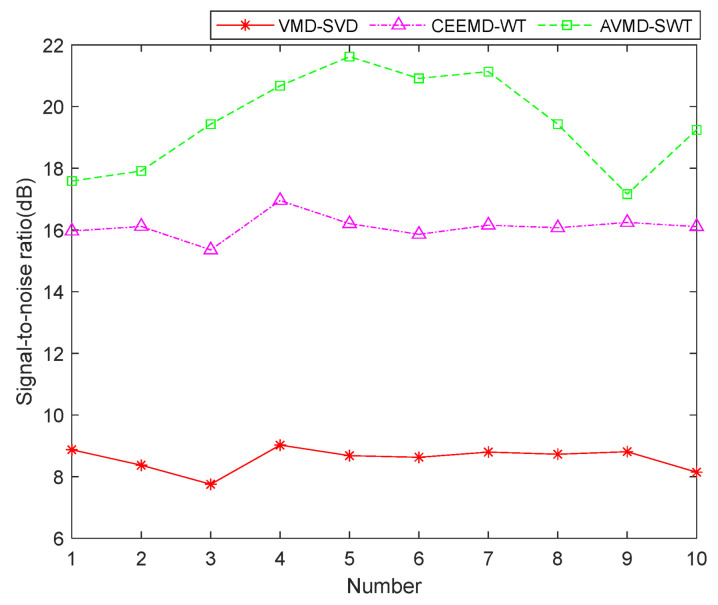
Signal-to-noise ratio of three noise reduction signals.

The signal denoised by VMD-SVD shows some overlap with the original signal, with a poorer performance in the low-frequency part, but slightly capturing the developmental trend of the original signal in the high-frequency part. CEEMD-WT demonstrates superior performance in comparison to VMD-SVD, as it is capable of capturing the signal trend across the entire frequency range, and the denoised signal fits the original signal curve well. However, there are still some noise components in the denoised signal, resulting in a moderate denoising performance. AVMD-SWT tightly fits the original signal curve in both the low-frequency and high-frequency parts. Compared to the previous two denoising algorithms, the denoised curve after AVMD-SWT is smoother, indicating the best denoising effect. Figure 4 shows the signal-to-noise ratio of the three denoised signals, demonstrating that the signal-to-noise ratio of AVMD-SWT is consistently higher than the other two algorithms over 10 cycles, indicating that AVMD-SWT achieves the best denoising performance.

### 2.2. ERCMFDE

#### 2.2.1. Multiscale Fluctuation Dispersion Entropy

As a nonlinear dynamic feature extraction method, MFDE can effectively measure regularities and detect mutations in vibration signals. Assume we have a univariate signal of length $L:u=\left\{u_{1},u_{2},\cdots ,u_{L}\right\}$. The original signal *u* is first divided into non-overlapping segments of length τ, named the scale factor. Then, the average of each segment is calculated to derive the coarse-grained signals as follows:

(14) $$x_{j}^{\left(\tau \right)}=\frac{1}{\tau }\sum_{b=\left(j-1\right)\tau +1}^{j\tau }u_{b},1\leq j\leq \left[\frac{L}{\tau }\right]=N$$

The FDE of the univariate time series of length $N:X=\left\{x_{1},x_{2},\cdots ,x_{N}\right\}$ is defined as follows [43]:

Step 1. The time series $X=\left\{x_{1},x_{2},\cdots ,x_{N}\right\}$ should be mapped onto the time series $Y=\left\{y_{1},y_{2},\cdots ,y_{N}\right\}$ by means of a normal distribution function.

(15) $$y_{i}=\frac{1}{\sigma \sqrt{2\pi }}\int_{-\infty }^{x_{i}}e^{\frac{-{\left(t-\mu \right)}^{2}}{2\sigma^{2}}}dt$$

where j=1,2,⋯,N; and σ and μ denote the standard deviation and mean value of time series *X*, respectively.

Step 2. Map the time series *Y* onto *c* classes. We map *Y* onto $Z^{c}=\left\{z_{1}^{c},z_{2}^{c},\cdots ,z_{N}^{c}\right\}$ by using $round\left(c\cdot y_{i}+0.5\right)$, where $z_{j}^{c}$ is the *jth* element of the classification sequence, *c* is the number of categories, and *round*() is the rounding function. 

Step 3. Phase space reconstruction: We compute the embedding vector based on the embedding dimension *m* and the time delay *d*. The elements of the embedding vector are defined as follows:

(16) $$z_{i}^{m,c}=\left[z_{i}^{c},z_{i+d}^{c},\cdots ,z_{i+\left(m-1\right)d}^{c}\right],i=1,2,\cdots ,N-\left(m-1\right)d$$

Step 4. Each series $z_{i}^{m,c}$ is mapped onto a pattern $\pi_{v_{o}v_{1}\cdots v_{m-1}}$ based on its values, while the following holds:

(17) $$z_{i}^{c}=\nu_{0},z_{i+d}^{c}=\nu_{1},\cdots ,z_{i+\left(m-1\right)d}^{c}=\nu_{m-1}$$

The number of fluctuation-based scattering patterns of $z_{i}^{m,c}$ is equal to ${\left(2c-1\right)}^{m-1}$.

Step 5. Calculate the probability corresponding to the scattering pattern $\pi_{v_{o}v_{1}\cdots v_{m-1}}$ with the following formula:

(18) $$p\left(\pi_{\nu_{0}\nu_{1}\cdots \nu_{m-1}}\right)=\frac{Num\left(\pi_{\nu_{0}\nu_{1}\cdots \nu_{m-1}}\right)}{N-\left(m-1\right)d}$$

where $Num\left(\pi_{\nu_{0}\nu_{1}\cdots \nu_{m-1}}\right)$ denotes the number of $z_{i}^{m,c}$ mapped onto $\pi_{v_{o}v_{1}\cdots v_{m-1}}$, i.e., the probability $p\left(\pi_{\nu_{0}\nu_{1}\cdots \nu_{m-1}}\right)$ is equal to the number of $z_{i}^{m,c}$ mapped onto $\pi_{v_{o}v_{1}\cdots v_{m-1}}$ divided by the total number of embedding vectors corresponding to the embedding dimension *m*.

Step 6. Calculate the FDE as follows:

(19) $$FDE\left(x,m,c,d\right)=-\sum_{\pi =1}^{{\left(2c-1\right)}^{m-1}}p\left(\pi_{\nu_{0}\nu_{1}\cdots \nu_{m-1}}\right)\cdot \ln\left[p\left(\pi_{\nu_{0}\nu_{1}\cdots \nu_{m-1}}\right)\right]$$

The FDE calculation takes into account the differences between the neighboring elements in the dispersion pattern. The FDE has a maximum value of $\ln\left({\left(2n-1\right)}^{m-1}\right)$ when all the fluctuating scattering patterns have the same probability value, resulting in an (m−1) dimensional pattern vector.

Thus, the normalized MFDE can be expressed as follows:

(20) $$MFDE=FDE\left(x_{j}^{\left(\tau \right)},m,c,d\right)$$

#### 2.2.2. Ensemble Refined Composite Multiscale Fluctuation Dispersion Entropy

In ERCMFDE, for a scale factor τ, τ different time series corresponding to the different starting points of the coarse-graining process are created. The $k^{th}$ coarse-grained time series $x_{k}^{\left(\tau \right)}=\left\{x_{k,1}^{\left(\tau \right)},x_{k,2}^{\left(\tau \right)},\cdots ,x_{k,j}^{\left(\tau \right)}\right\}$ of *u* at scale factor τ can be calculated by using three different methods to obtain multiple coarse-grained series. The three different methods can be expressed as follows:

(21) $$x_{k,j}^{\left(\tau \right)}{|}_{\mathrm{mean}}=\frac{1}{\tau }\sum_{b=k+\tau (j-1)}^{k+\tau j-1}u_{b},\;1\leq j\leq N,\;1\leq k\leq \tau$$

(22) $$x_{k,j}^{\left(\tau \right)}{|}_{\mathrm{var}}=\frac{1}{\tau }{\sum_{b=k+\tau (j-1)}^{k+\tau j-1}\left(u_{b}-{\bar{u}}_{b,j}\right)}^{2},\;1\leq j\leq N,\;1\leq k\leq \tau$$

(23) $$x_{k,j}^{\left(\tau \right)}{|}_{skewness}=\frac{1}{\tau }{\sum_{b=k+\tau (j-1)}^{k+\tau j-1}\left(u_{b}-{\bar{u}}_{b,j}\right)}^{3},\;1\leq j\leq N,\;1\leq k\leq \tau$$

where ${\bar{u}}_{b,j}=\frac{1}{\tau }\sum_{b=k+\tau (j-1)}^{k+\tau j-1}u_{b}$.

For each coarse-grained series, the RCMFDE can be defined as follows [44]:

(24) $$RCMFDE(x_{k},m,c,d,\tau )=-\sum_{\pi =1}^{{\left(2c-1\right)}^{m-1}}{\bar{p}}^{\tau }\left(\pi_{\nu_{0}\nu_{1}\cdots \nu_{m-1}}\right)\cdot \ln\left[{\bar{p}}^{\tau }\left(\pi_{\nu_{0}\nu_{1}\cdots \nu_{m-1}}\right)\right]$$

where ${\bar{p}}^{\tau }\left(\pi_{\nu_{0}\nu_{1}\cdots \nu_{m-1}}\right)=\frac{1}{\tau }\sum_{k=1}^{\tau }p_{k}^{\tau }$, and $p_{k}^{\tau }$ is the probability of each possible scattering pattern $\pi_{\nu_{0}\nu_{1}\cdots \nu_{m-1}}$ for the different starting points.

## 3. The Proposed Intelligent Gearbox Fault Diagnosis Method

### 3.1. RFE

**Figure 5:**
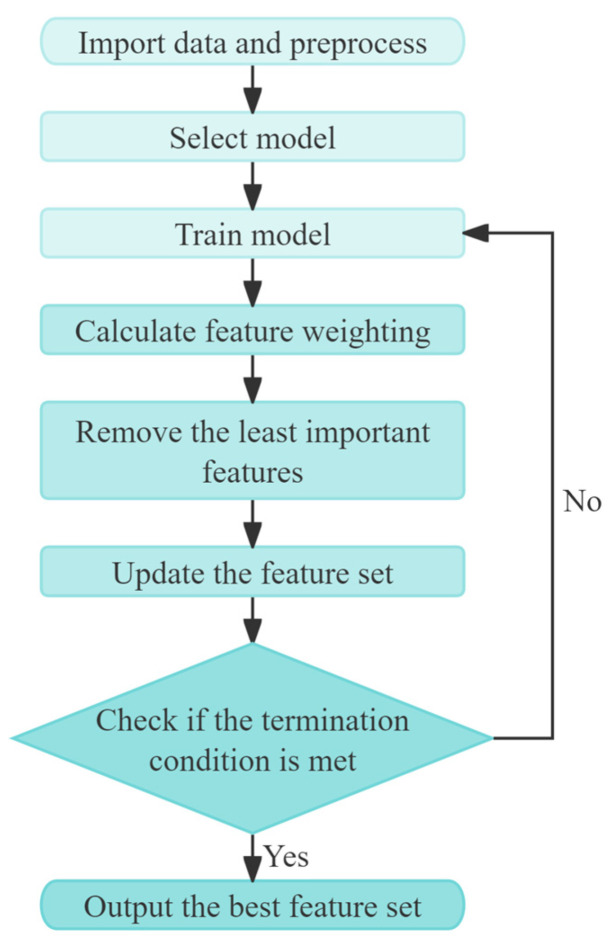
The flowchart of the RFE algorithm.

It is typical for raw data to include a significant number of superfluous features. The presence of these superfluous redundant features may result in the overfitting of the algorithmic model, thereby impairing the algorithm’s capacity for generalization. Consequently, the features are typically subjected to preprocessing in order to enhance model performance and reduce computational costs. RFE is a method for selecting the optimal subset of features [45]. It involves constructing a model, typically a tree-based model such as a decision tree or random forest, and then progressively eliminating the least important features based on the feature importance of the model. RFE can be utilized in conjunction with a multitude of assessment methodologies and models, exhibiting a high degree of flexibility and applicability. Its performance in feature selection has been demonstrated to be stable and reliable. Figure 5 shows the flowchart of the RFE algorithm.

### 3.2. SSA-SVM

The Sparrow Search Algorithm is a heuristic optimization algorithm based on the foraging behavior of sparrows in nature, aiming to find the optimal solution by simulating the foraging strategy of sparrows [46]. The SSA is distinguished by its simplicity, ease of implementation, and high efficiency, which collectively demonstrate its effectiveness for solving optimization problems. A Support Vector Machine is a classic machine learning algorithm used primarily for tasks such as pattern recognition, classification, and regression [47].

The Sparrow Search Algorithm is employed to explore the parameter space and minimize the objective function. In order to identify the optimal solution, the SSA employs a process of simulation, which emulates the foraging behavior of sparrows. This encompasses a range of activities, including foraging, communication, and migration strategies. The optimization results are analyzed, and the performance of the SVM after parameter optimization is evaluated. The Sparrow positions are used to represent the penalty parameter *c* and the kernel parameter *g* of the SVM. Through sorting the global fitness values, the optimal value and optimal position are determined to obtain the optimal parameters. The specific algorithm of the SVM can be found in reference [48].

### 3.3. The Proposed Fault Diagnosis Scheme

**Figure 6:**
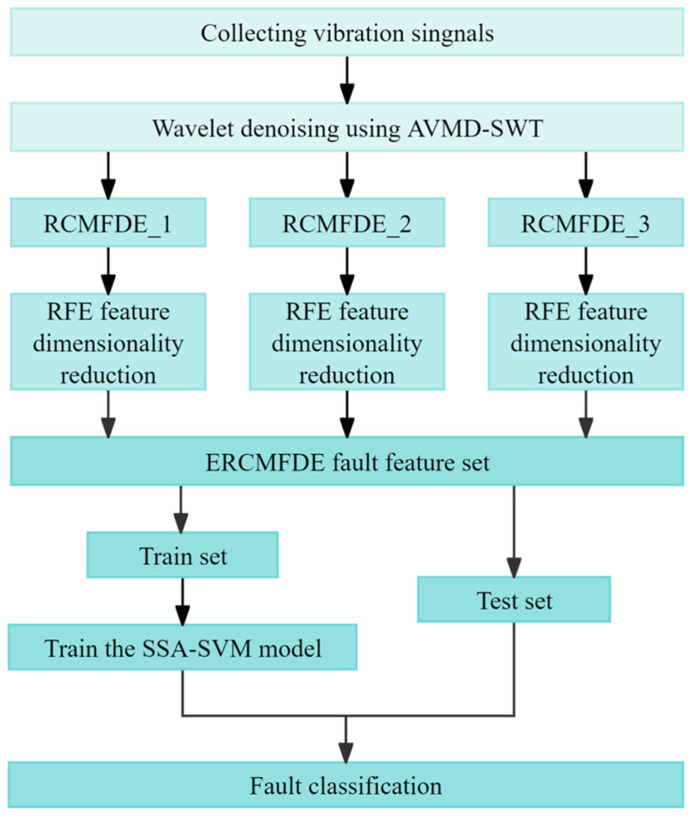
Fault diagnosis flowchart based on ERCMFDE.

This section proposes a gear fault diagnosis method based on the ERCMFDE, combining AVMD-SWT, RFE, and SSA-SVM, with the aim of overcoming the limitations of a single tension fault feature extraction. The proposed methodology is elucidated in Figure 6, which depicts the fault diagnosis flowchart. The specific steps are as follows:

Step 1. Signal acquisition and preprocessing: an acceleration sensor is employed to collect the vibration data during the operation of the gearbox, and AVMD-SWT is utilized to adaptively decompose and reconstruct the vibration signal, thereby effectively filtering out the noise.

Step 2. Signal segmentation: the long signal is divided into N sub-signals of length 2048, in accordance with the principle of equidistant sampling.

Step 3. Feature extraction: the ERCMFDE is employed to extract the features from the sub-signal set, thereby obtaining a subset of fault features based on three different moments.

Step 4. Feature dimensionality reduction and selection: Each feature in the subset of features is subjected to dimensionality reduction using a recursive feature elimination algorithm, and three features are selected from each of them. The selected features thus constitute a new fault feature set.

Step 5. Model training and testing: The fault feature set is randomly divided into a training set and a testing set in accordance with the specified ratio. Subsequently, the model is trained using the SSA-SVM algorithm. Finally, fault identification is performed on the test set.

## 4. Experimental Verification

### 4.1. Data from MFS

#### 4.1.1. Description and Division of Data

**Figure 7:**
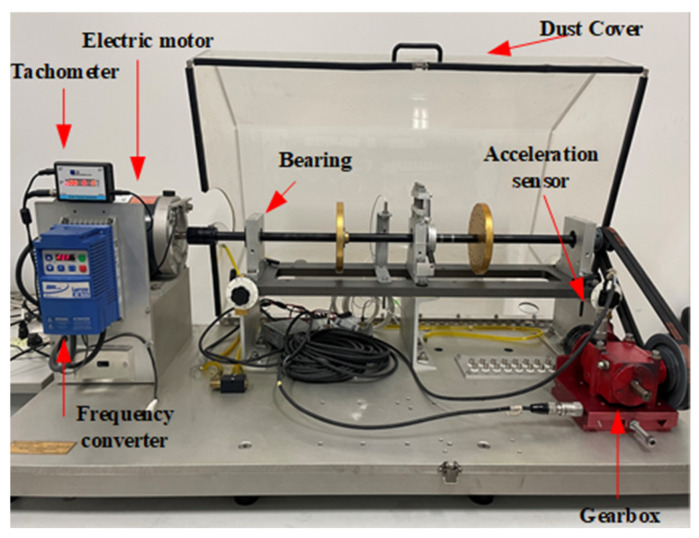
MFS experiment system.

**Figure 8:**
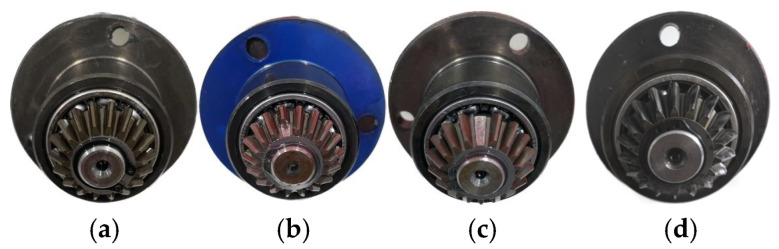
Four gear states: (**a**) normal state; (**b**) broken tooth fault; (**c**) missing tooth fault; and (**d**) surface wear fault.

The gearbox dataset is provided by a mechanical failure simulation system (MFS), which is manufactured by SQI with the objective of precisely simulating a wide range of common mechanical device operating conditions. The experimental bench is constructed with modular components and is both powerful and reliable. The system can be employed for the simulation and analysis of common failures of bearings and gears in transmission systems in the energy sector. As illustrated in Figure 7, the principal component of the experimental system is an integrated mechanical failure simulation test bench and data acquisition apparatus. As illustrated in Figure 8, the gearbox fault diagnosis research kit utilized in the experiment comprises a variety of faults, including missing gears, broken gears, and gear tooth wear.

The experiments were conducted with the motor operating at no load, and the normal state and three fault states were tested. The original samples were divided into 100 subsamples, each with a length of 2048. The four distinct operational states of the gear, which encompass both nominal and faulty conditions, are illustrated in Figure 8.

The experimental system was constructed to include a drive motor, bearing assembly, and operating gearbox, with the motor having a high-speed capability of up to 3600 revolutions per minute. Furthermore, the output of the gearbox was connected to an adjustable magnetic brake mechanism that allowed the operator to intuitively control the load conditions through a rotary operation over a range of torque values, identified by a dial scale from 0 to 5. The experiments were conducted utilizing accelerometers that were meticulously positioned on the gearbox and its base in order to capture the vibration signals at a sampling rate of 12 kHz. The experimental platform was capable of not only supporting an in-depth exploration of the vibration characteristics of a single equipment failure, but also of revealing the complex interactions and coupling effects between multiple failures. This was accomplished by employing a flexible approach to combining different failure simulation modules within a simulation environment that modeled the operation of a mechanical system.

#### 4.1.2. Signal Denoising

**Figure 9:**
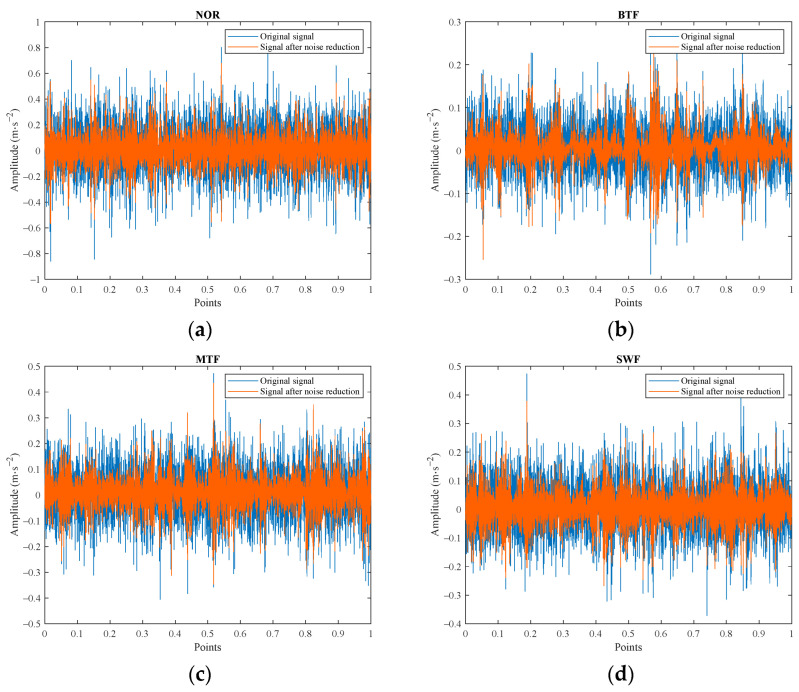
Original signal and noise reduction signal in four gear states: (**a**) NOR; (**b**) BTF; (**c**) MTF; and (**d**) SWF.

By collecting the vibration signals of the four types of faulty gears under no-load conditions, with the motor averaging 1750 revolutions per minute, and subjecting them to AVMD-SWT denoising processing, the temporal waveforms can be observed, as shown in Figure 9. In the normal state (NOR), the vibration signals exhibit stability, periodicity, and relatively small amplitudes, presenting an overall continuous and regular waveform. However, the vibration signal resulting from the broken tooth fault (BTF) exhibits noticeable impulsiveness in the temporal graph, characterized by sudden increases and decreases in amplitude, due to the abrupt transmission mutation between the gears at the point of the broken teeth. In comparison, the temporal waveform of the missing tooth fault (MTF) is more pronounced, displaying stronger impulsiveness and clearer periodicity. Additionally, gear surface wear, also known as a surface wear fault (SWF), results in geometric alterations, exacerbating the presence of high-frequency components in the vibration signal.

#### 4.1.3. Feature Extraction

The MFS dataset comprises the operational data of the gearbox under the four states, with each fault type consisting of 100 samples with a data length of 2048, forming the fault sample set, as detailed in Table 1. Under the following parameters, with the embedding dimension *m* as 2, the classification category c = 5, the scale factor *s* as 20, and the time delay *d* as 1 [49], the RCMFDE_1, RCMFDE_2, and RCMFDE_3 values of each signal in the sample set are computed, resulting in three error feature matrices of size 400 × 20, 400 × 19, and 400 × 18, where 400 represents the number of samples and 20, 19, and 18 indicate the scale size.

Following the refined composite multiscale dispersion entropy method previously introduced for the feature extraction of gearbox vibration fault signals, the following eigenvalues are obtained: RCMDE_1, RCMRDE_1, and RCMFDE_1 processed by the first-order center-moment method; RCMDE_2, RCMRDE_2, and RCMFDE_2 processed by the second-order center-moment method; RCMDE_3, RCMRDE_3, and RCMFDE_3 processed by the third-order center-moment method.

**Figure 10:**
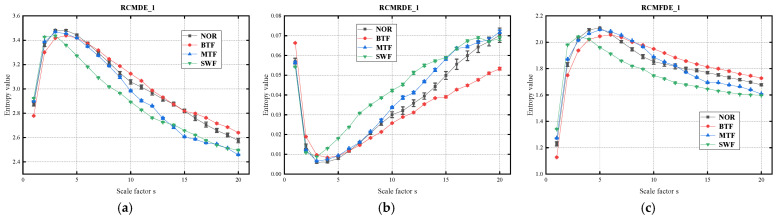

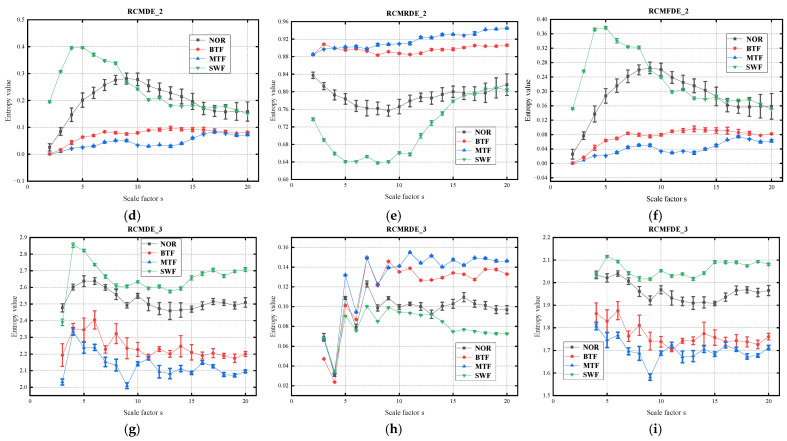
Entropy values: (**a**) RCMDE_1; (**b**) RCMRDE_1; (**c**) RCMFDE_1; (**d**) RCMDE_2; (**e**) RCMRDE_2; (**f**) RCMFDE_2; (**g**) RCMDE_3; (**h**) RCMRDE_3; and (**i**) RCMFDE_3.

Figure 10 illustrates the standard deviations of the various coarse-graining techniques for the refined composite multiscale dispersion entropy (RCMDE), refined composite multiscale reverse dispersion entropy (RCMRDE), and RCMFDE, as calculated for the four fault categories. A comparison of the differences between the dispersion entropy, reverse dispersion entropy, and fluctuation dispersion entropy reveals that each has distinct advantages in different coarse-graining theories. In RCMDE_2, effective differentiation is demonstrated at smaller scale factors. In RCMRDE_3, the error bars are relatively small and the stability of the entropy values is high. However, when the RCMFDE is considered in isolation, there is a notable enhancement in its discriminatory capacity, progressing from first-order central moments to second-order center distance to third-order center distance. In RCMFDE_1, the error bars are relatively small, indicating a high degree of stability in the entropy values and an effective discrimination between scales 13 and 20. Conversely, RCMFDE_2 demonstrates a high discrimination at smaller scale factors; however, at larger scale factors, it becomes challenging to distinguish between healthy gear vibration signals and signals indicating tooth wear faults. In RCMFDE_3, the entropy curves of the majority of fault states exhibit notable differences, thereby demonstrating a marked enhancement in discriminative ability. Overall, the ERCMFDE-based feature extraction employs a diverse range of features to extract comprehensive feature information from time series data, thereby offering a more comprehensive approach to fault feature extraction than single fine-scale composite multiscale entropy extraction methods.

#### 4.1.4. Feature Reduction and Visualization

After extracting the fault features for all types of faults using the ERCMFDE, we obtained a feature set of size 400 × 20 in RCMFDE_1, a feature set of size 400 × 19 in RCMFDE_2, and a feature set of size 400 × 18 in RCMFDE_3. Unsupervised feature selection was performed using RFE to rank the importance of the features in each feature set. The top 3 most important features were selected from each feature set to form a new fault feature set of size 400 × 9. This feature set was then divided into training and testing samples.

**Figure 11:**
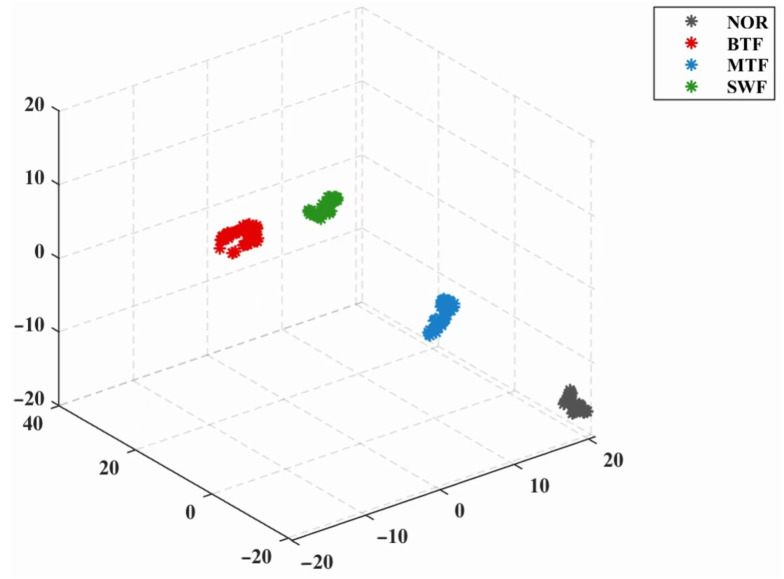
The 3D result obtained using the t-SNE.

The t-SNE algorithm was applied to the training set feature matrix, and a high-dimensional-to-low-dimensional conversion was performed to reduce the size of the training set feature matrix to 240 × 9. The resulting data were represented in a three-dimensional graph. As illustrated in Figure 11, the four fault states are discernible and distinct from one another. The 3D visualization outcome aligns with the intended outcome, indicating that the t-SNE algorithm is capable of effectively extracting pivotal information from high-dimensional features, thereby facilitating the differentiation of gearbox states. 

#### 4.1.5. Feature Selection and Result Analysis

**Figure 12:**
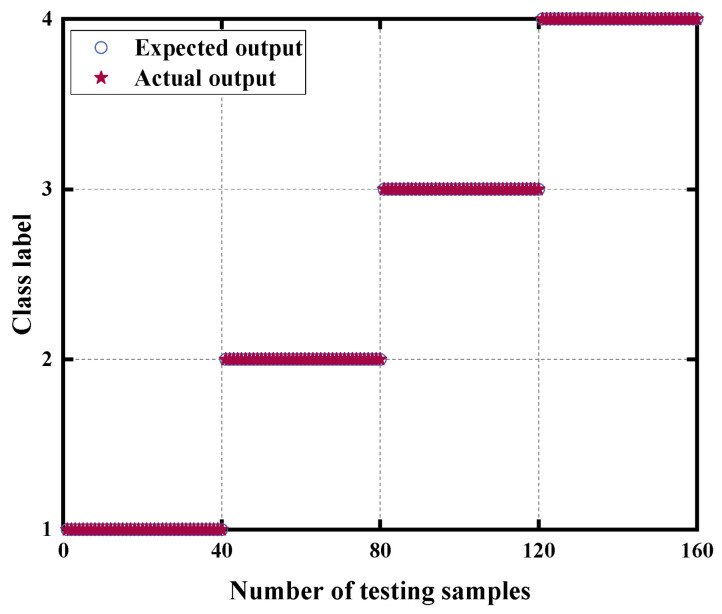
SSA-SVM classification results.

The training samples are employed to train the SSA-SVM parameters, and the trained SSA-SVM is utilized to classify the test samples into faults. The results of the fault diagnosis are illustrated in Figure 12, demonstrating 100% fault diagnosis accuracy and no misclassified samples.

### 4.2. Experimental Comparison

#### 4.2.1. Different Moments of Entropy

**Figure 13:**
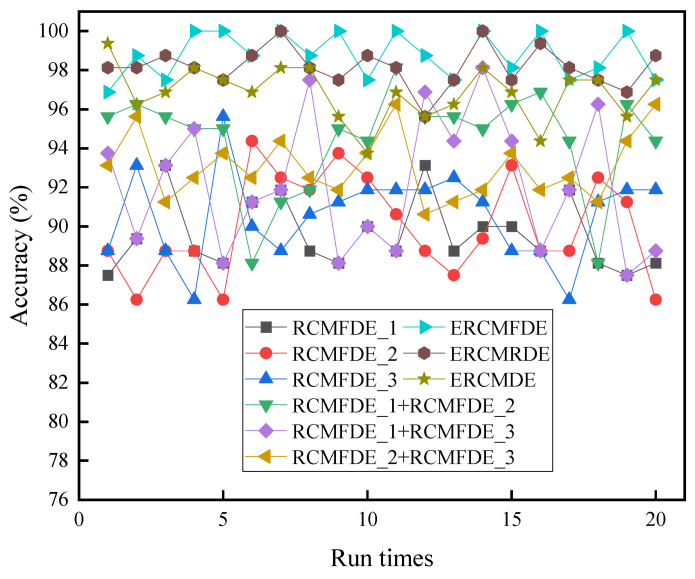
Accuracy of nine algorithms for troubleshooting.

In order to compare the efficacy of the disparate feature extraction algorithms and to ascertain the advantages of the ERCMFDE for the comprehensive extraction of signal features, 20 repeated experiments were conducted utilizing the same fault sample data and feature selection method. The efficacy of the various feature extraction algorithms was evaluated through the application of RCMFDE_1, RCMFDE_2, RCMFDE_3, and their pairwise combinations, as well as the RCMDE and RCMRDE. Table 2 depicts the mean fault diagnosis accuracy of the nine feature extraction algorithms, while Figure 13 illustrates the fault diagnosis accuracy.

As evidenced in Table 2, the ERCMFDE algorithm, which employs a comprehensive signal feature extraction approach, exhibits the highest fault diagnosis accuracy, reaching 100%. The average diagnosis accuracy is superior to that of the remaining eight algorithms. Compared to the first-order central-moment RCMFDE_3, which boasts the highest average accuracy, the ERCMFDE demonstrates an improvement of 8.22%. A comparison of the pairwise combinations of the first-, second-, and third-moment scale entropy methods reveals that the average accuracy of the ERCMFDE is increased by 4.34%, 6.59%, and 5.72%, respectively. Furthermore, upon considering the various multiscale fine composite entropy methods, the ERCMFDE’s average accuracy surpasses that of ERCMDE by 1.94% and ERCMRDE by 1.31%. The experimental results provide evidence to support the claim that integrating a multiscale fluctuation dispersive entropy algorithm with multiple coarse-graining processes can enhance the efficiency of information acquisition for feature extraction, thereby addressing the limitations of the RCMFDE in this domain.

#### 4.2.2. Different Entropy Algorithms

We compare the RCMFDE with widely used entropy-based feature extraction methods to demonstrate the superiority of the FDE method. We compare it with the sample entropy (SE), fuzzy entropy (FE), permutation entropy (PE), DE, and RDE. The relevant parameters are chosen as follows: *m* = 2, *c* = 5, *d* = 1, *s* = 20, *n* = 2, *r* = 0.15 SD [49,50,51,52,53,54].

**Figure 14:**
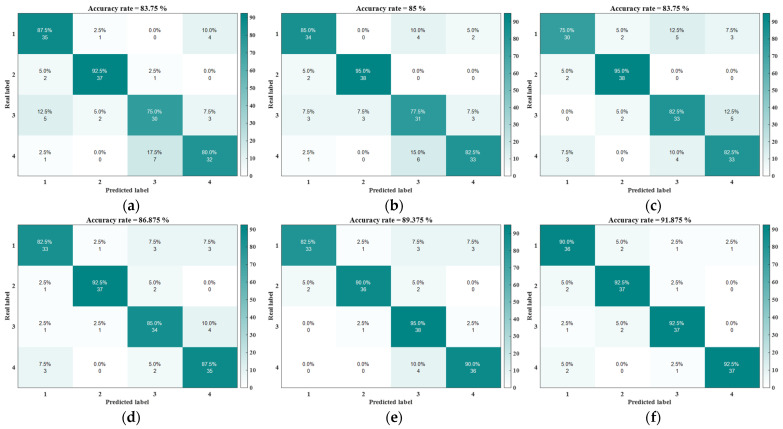
Confusion matrix: (**a**) RCMSE; (**b**) RCMFE; (**c**) RCMPE; (**d**) RCMDE; (**e**) RCMRDE; and (**f**) RCMFDE.

After the refined composite multiscaling of the first-order moments using the above entropy method, the feature set after AVMD-SWT noise reduction is extracted and the feature matrix is input into the SSA-SVM classification model for the training and prediction of the classification results. The classification results of the different entropy algorithms are shown in the confusion matrix in Figure 14. Each group of comparison experiments was repeated 25 times and the average accuracy of the fault diagnosis, as well as the maximum and minimum accuracy values, are shown in Table 3.

As shown in Table 3, the RCMFDE algorithm has the highest fault diagnosis accuracy of 96.25%. In addition, its average diagnosis accuracy is also better than that for the remaining five entropy algorithms. The experimental results prove that the fluctuation-dispersed entropy algorithm is more effective for a time series problem, more sensitive to a gearbox’s state information, and can characterize a gearbox fault signal more effectively.

#### 4.2.3. Different Classification Methods

We compare the SSA-SVM with widely used intelligent classification models to demonstrate the superiority of the proposed model. We compare it with decision tree (DT), Extreme Learning Machine (ELM), SSA-ELM, SVM, and Genetic Algorithm-optimized SVM (GA-SVM).

**Figure 15:**
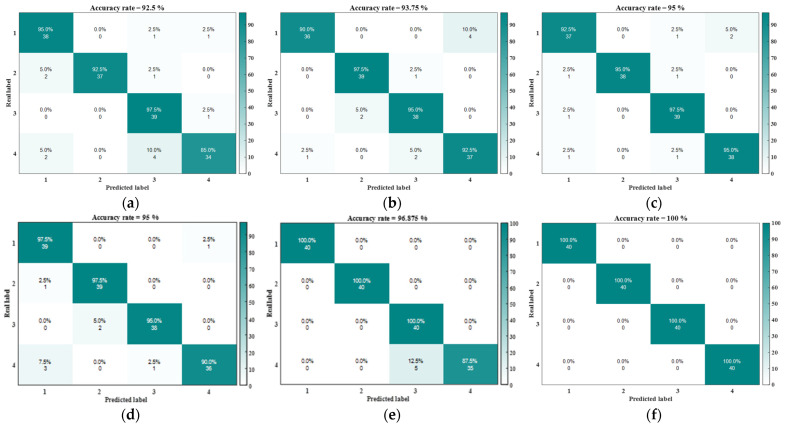
Confusion matrix: (**a**) DT; (**b**) ELM; (**c**) SSA-ELM; (**d**) SVM; **(e**) GA-SVM; and (**f**) SSA-SVM.

The feature set after AVMD-SWT noise reduction is extracted by the ERCMFDE proposed in this paper, and the feature matrix is input into the different intelligent classification models for the training and predicting of the classification results. The classification results of the different classification models are shown in the confusion matrix in Figure 15. Each group of comparison experiments was repeated 25 times, and the average accuracy of the fault diagnosis, as well as the maximum and minimum accuracy values, are shown in Table 4.

As shown in Table 4, the SSA-SVM classification model has the highest fault diagnosis accuracy of 100%. In addition, the average diagnosis accuracy is also better than that of the remaining five classification models. The experimental results show that the proposed SSA-SVM is able to handle datasets with a large number of features in a high-dimensional space with excellent classification results.

#### 4.2.4. Noise Resistance Test

In practical engineering applications, the data collected by vibration acceleration sensors installed in gearboxes frequently exhibit a considerable degree of noise. The aim of our experiments is to investigate the efficacy of the model in diagnosing gearbox faults in the presence of background noise. This will enable us to ascertain the robustness of the model and to verify its practicality and anti-interference ability in a real environment.

**Figure 16:**
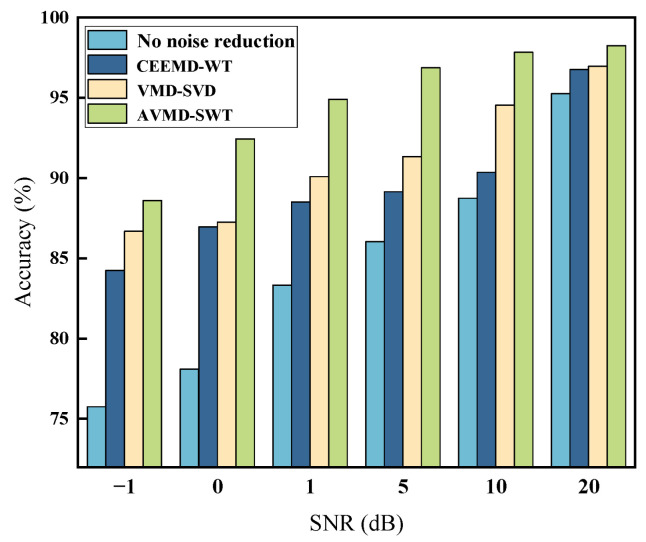
Classification results of different models with noise content.

Gaussian white noise with varying levels of interference is introduced into the data set in order to achieve different signal-to-noise ratios (SNRs). In order to validate the performance of the AVMD-SWT model proposed in this study with regard to noise immunity, five sets of noisy signals were used as the experimental data set. The control benchmark models for the comparison experiments were VMD-SVD, CEEMD-WT, and no noise reduction. Figure 16 shows the average of the diagnostic accuracy for 25 repetitions of the experiment.

The diagnostic classification accuracy of the other noise reduction models is significantly reduced in the presence of elevated noise levels. It can be concluded that the AVMD-SWT model proposed in this study demonstrates superior anti-noise performance in comparison to the other models.

## 5. Conclusions

This paper proposes an intelligent fault diagnosis model for gearboxes based on AVMD-SWT and ERCMFDE, targeting the characteristics of gearbox motion. The results of an experimental validation exercise demonstrate the superiority of this model in terms of signal processing and feature extraction. The conclusions obtained are as follows:

1. The AVMD-SWT algorithm outperforms VMD-SVD and CEEMD-WT in signal decomposition accuracy and the suppression of mode mixing. To address the influence of the parameter selection on the VMD, we propose the AVMD, which combines kurtosis and autocorrelation coefficients to determine the number of decomposition layers. Additionally, we integrate an SWT to denoise the noisy mode components. The efficacy of this denoising method for analyzing gearbox vibration fault signals is validated through both simulated signals and MFS experimental signals.
2. We extend the coarse-graining method of the first-order central moment to include three approaches: the first-order central moment, the second-order central moment, and the third-order central moment. This extension forms the basis of the ERCMFDE algorithm for feature extraction from fault signals. Recognizing the limitations of coarse-graining solely based on the first-order central moment in feature extraction, this method aims to enrich the representation of fault signals by employing multiple perspectives for the coarse-graining of vibration signals.
3. Combining the strengths of recursive feature elimination and the Sparrow Search Algorithm–Support Vector Machine in the feature selection for fault diagnosis, we propose an intelligent fault diagnosis model for gearboxes based on AVMD-SWT, ERCMFDE, RFE, and SSA-SVM.
4. The model is validated using the MFS comprehensive fault experiment platform, demonstrating the superiority of the ERCMFDE over a single entropy feature. It provides more comprehensive feature extraction and achieves a high diagnostic accuracy of 98.78%.

## Figures and Tables

**Table 1:** Description of MFS data.

Fault Types	Motor Speed (r/min)	Number of Training Samples	Number of Testing Samples	Class Label
Normal	1750	60	40	1
Broken tooth fault	1750	60	40	2
Missing tooth fault	1750	60	40	3
Surface wear fault	1750	60	40	4

**Table 2:** Fault diagnosis accuracy of nine kinds of algorithms.

Entropy	Minimum Accuracy %	Maximum Accuracy %	Average Accuracy %
RCMFDE_1	87.50	93.13	89.59
RCMFDE_2	86.25	94.38	90.03
RCMFDE_3	86.25	95.63	90.56
RCMFDE_1 + RCMFDE_2	88.13	98.13	94.44
RCMFDE_1 + RCMFDE_3	87.50	98.13	92.19
RCMFDE_2 + RCMFDE_3	90.63	96.25	93.06
ERCMDE	93.75	99.38	96.84
ERCMRDE	95.63	100.00	97.47
ERCMFDE	96.88	100.00	98.78

**Table 3:** Accuracy of different entropy algorithms.

Entropy	Minimum Accuracy %	Maximum Accuracy %	Average Accuracy %
RCMSE	78.13	87.50	83.56
RCMFE	82.50	93.75	88.44
RCMPE	83.75	91.25	87.94
RCMDE	82.50	93.75	89.19
RCMRDE	87.50	95.63	89.44
RCMFDE	88.13	96.25	91.50

**Table 4:** Accuracy of different classification methods.

Classification Method	Minimum Accuracy %	Maximum Accuracy %	Average Accuracy %
DT	91.25	97.50	94.56
ELM	92.50	97.50	93.75
SSA-ELM	93.75	99.38	96.44
SVM	91.25	97.50	95.19
GA-SVM	92.50	100.00	95.31
SSA-SVM	96.25	100.00	98.00

## Data Availability

The data presented in this study are available on request from the corresponding author.
